# A phase 1b randomised clinical trial evaluating BBI-001, a non-absorbed oral therapeutic for the treatment of iron overload

**DOI:** 10.1038/s41598-025-01421-4

**Published:** 2025-05-17

**Authors:** Curtis Scribner, Jamie Cope, Philip Ryan, John K. Olynyk, John Ryan, J. Daniel Griffin, Cory Berkland

**Affiliations:** 1 Bond Biosciences, Inc., Parkville, MO USA; 2 POG Consulting, Inc., Berkeley, CA USA; 3 Nucleus Network, Melbourne, VIC Australia; 4 Curtin Medical School, Curtin University, Bentley, WA Australia; 5 Department of Gastroenterology & Hepatology, Fiona Stanley Fremantle Hospital Group, Murdoch, WA Australia; 6 Hepatology Unit, Beaumont Hospital/RCSI University of Medicine and Health Sciences, Dublin, Ireland; 7 Department of Biomedical Engineering, Washington University in St. Louis, St. Louis, MO USA; 8 Department of Chemistry, Washington University in St. Louis, St. Louis, MO USA

**Keywords:** Haematological diseases, Molecular medicine

## Abstract

Non-transfusion-dependent iron overload is the result of excessive dietary iron absorption, most commonly caused in populations of European descent by the genetic disorder HFE-related hemochromatosis (HH). In this disorder, hyperabsorption of 3–5 mg of iron per day cannot be counterbalanced by the typical passive elimination of 1–2 mg of iron each day into the feces by the shedding of enterocytes. Therefore, the current standard of care for most HH individuals who develop iron overload is to undergo systemic iron reduction with induction-phase phlebotomy therapy followed by long-term maintenance phlebotomy therapy. Unfortunately, long-term compliance with a regular phlebotomy regimen is less than 25% in some clinical settings. BBI-001 is a non-absorbed, oral therapeutic that binds dietary iron in the gut, preventing absorption and promoting iron elimination in the feces. The safety and efficacy of BBI-001 was confirmed in a single ascending dose, double-blind, Phase 1b clinical trial NCT05238207 (14/02/2022) in patients with iron deficiency. No treatment-related adverse events occurred for single doses of up to 2000 mg of BBI-001. The study also established proof-of-mechanism since BBI-001 significantly reduced the absorption of iron isotopes from breakfast meals compared to placebo. BBI-001 was most effective in subjects who hyperabsorbed iron (> 3 mg) on placebo, suggesting an ability to normalize iron absorption in at-risk patients. This study supports the further evaluation of BBI-001 as a safe pharmaceutical alternative to lifelong therapeutic phlebotomy.

**Supplementary Information:**

The online version contains supplementary material available at 10.1038/s41598-025-01421-4.

## Introduction

**Fig. 1:**
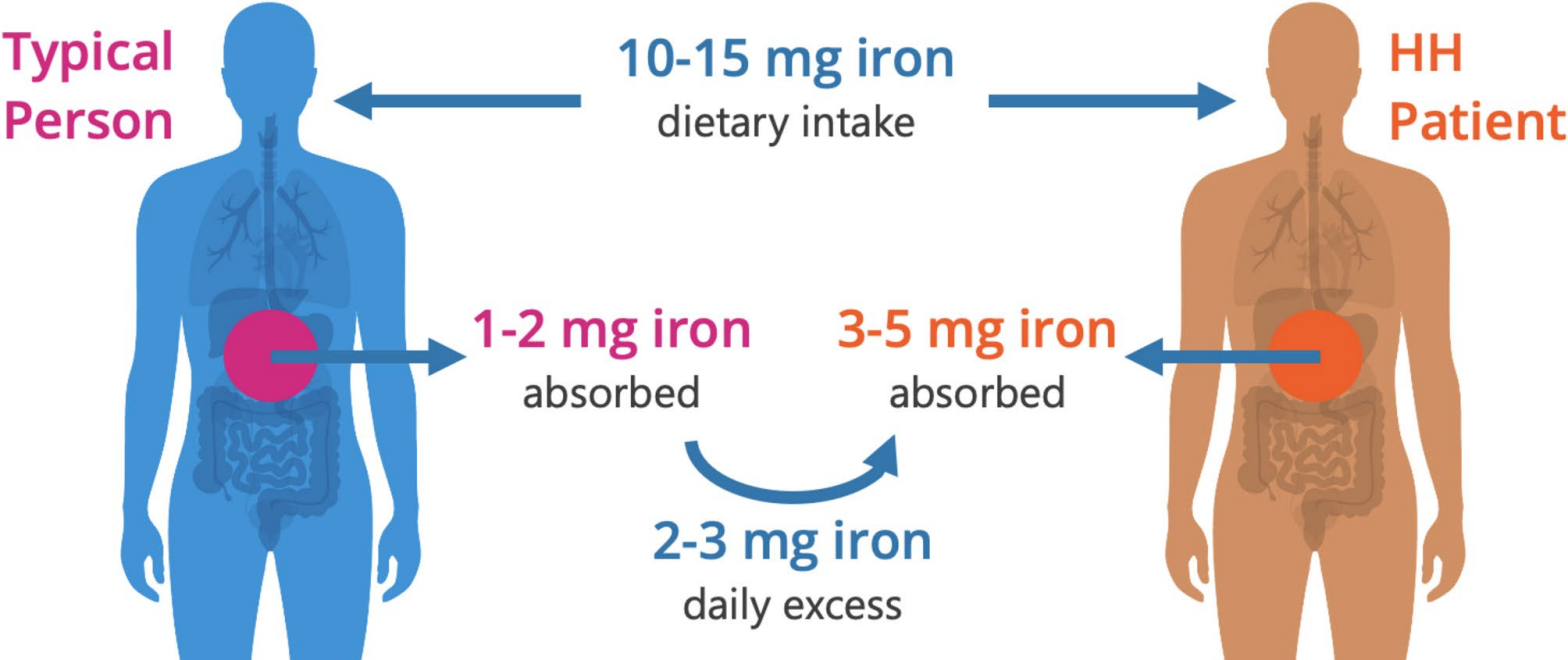
Iron mass balance. Typical healthy individuals (left) regulate iron balance while patients with HH (right) do not. Figure created using Microsoft 365 Powerpoint.

Iron overload can occur as a result of multiple blood transfusions or due to uncontrolled iron accumulation from the diet^1–3^. The latter is referred to as non-transfusion-dependent iron overload and is most commonly found in populations of European descent with the genetic disorder HFE-related hemochromatosis (HH). HH is a common genetic disorder with impaired production of hepcidin, the key iron-regulatory hormone. Normally, hepcidin limits the absorption of dietary iron to 1–2 mg per day. Up to 40% of subjects with HH may absorb in excess of 3–5 mg of iron per day and develop significant iron overload as a result of dysfunctional hepcidin. Iron can only be passively eliminated through tissue loss (i.e., bleeding/menstruation, sloughing of the intestinal lining) at a rate of ~ 1–2 mg per day. Thus, chronic hyperabsorption of dietary iron leads to iron overload (Fig. 1). Excess iron is stored in tissues, especially the pancreas, skin, liver, and heart, where it can ultimately cause significant organ damage from the production of reactive oxygen species by several different mechanisms^4^. If HH is diagnosed early and treatment to reduce the amount of iron in the blood is implemented before serious complications develop, then people living with HH can have a normal life expectancy. Delayed diagnosis or noncompliance with treatment may lead to irreversible organ damage leading to a reduced life expectancy, especially for those who develop hepatic cirrhosis^5–7^.

The typical current treatment for HH is therapeutic phlebotomy where blood is withdrawn on a regular basis to reduce whole-body iron content and prevent further tissue damage^8^. Chelation therapy is utilized when phlebotomy is not possible or contraindicated but is avoided due to drug toxicity. The American Association for the Study of Liver Diseases clinical practice guideline recommends maintaining serum ferritin at 50 to 100 ng/mL and reducing transferrin saturation levels to < 50% for males and < 45% for females^9^. Therapeutic phlebotomy for HH consists of two phases of treatment: induction and maintenance. Following diagnosis, patients may present with serum ferritin levels of 1000 ng/mL or higher, indicating a significant excess iron in tissues. The initial induction phase entails the removal of 1 unit (400–500 mL) of blood every 1–2 weeks for 12–18 months until serum ferritin levels are decreased to normal physiological levels (with a target of 50 to 100 ng/mL). Each phlebotomy removes about 200–250 mg of iron from the body and generally reduces the ferritin level by 30 to 50 ng/mL^10^. After induction, a typical patient would be prescribed maintenance phlebotomy every quarter (4 times per year) for the rest of their lives^11^.

Chronic hyperabsorption of iron through the gut in HH drives the need for lifelong maintenance phlebotomy. Diets that are low in iron are very difficult to achieve, have a marginal or variable effect, and are not recommended as a treatment for HH^12^. BBI-001 was developed to address the underlying mechanism of iron accumulation in HH by reducing the amount of dietary iron available for absorption. BBI-001 is a nonabsorbed, particulate formulation of a high-avidity iron binding moiety, dihydroxybenzoic acid (DHBA), conjugated to a crosslinked polyallylamine (PAAm) backbone. The crosslinked polymer emulates the naturally occurring structure of enterobactin, the most potent and selective iron chelator known^13,14^. BBI-001 has a particle size large enough to prevent systemic absorption and restrict exposure to the gastrointestinal tract^15^. BBI-001 possesses high iron specific avidity and blocks the absorption of dietary iron in animal models of iron-overload^16^.

A double-blind, placebo-controlled, Phase 1b, clinical trial was conducted to establish the safety of single escalating doses of BBI-001 and to establish proof-of-mechanism. Since BBI-001 is not absorbed from the gut after oral administration and is eliminated in the feces, the pharmacodynamic effect on iron absorption was assessed. Subjects were randomized to receive either BBI-001 or placebo in a crossover design. After overnight fasting, a standardized breakfast was supplemented with 10 mg of ^58^Fe and administered with placebo or 10 mg of ^57^Fe and administered with BBI-001. Blood was drawn over 24 h following each dose to evaluate the ability of BBI-001 inhibit iron absorption compared to placebo.

## Materials and methods

### Clinical

**Fig. 2:**
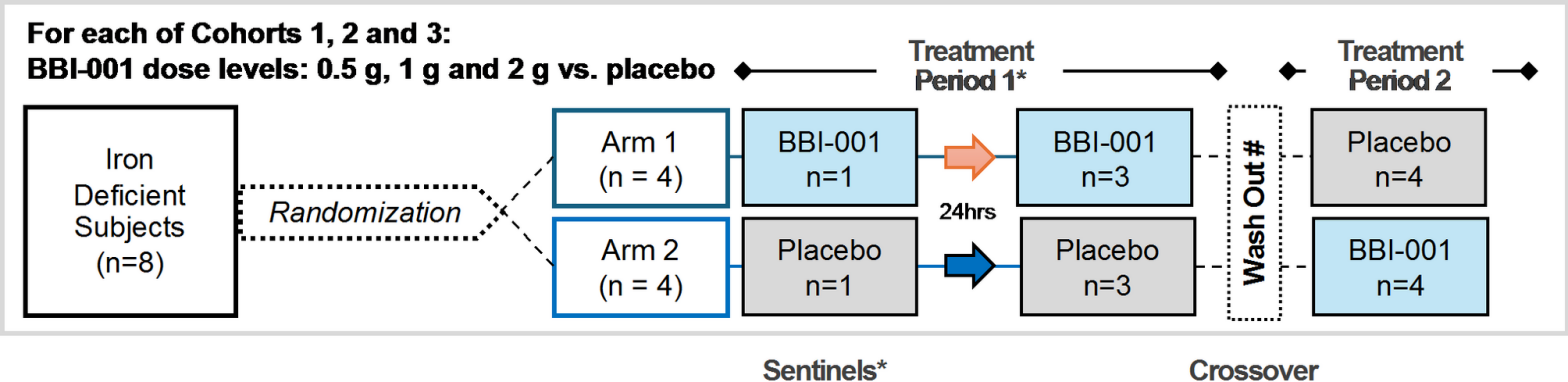
Trial design. This was a double blind, placebo controlled, intrasubject cross over, single ascending dose escalation study. Four subjects with iron deficiency were enrolled in each cohort and randomized 3:1 to receive BBI-001 or Metamucil placebo. Each subject received BBI-001 or placebo on Day 1 and then returned at least 1 week later to receive the alternate treatment. After at least 8 h of fasting, each subject received a low iron breakfast supplemented with Fe^57^ if given BBI-001 or Fe^58^ if given placebo.

A first-in-human, double-blind, randomized, placebo-controlled, two-arm crossover study was conducted to evaluate the safety, tolerability, and pharmacodynamics of ascending dose levels of BBI-001 after a single administration in iron deficient participants (NCT05238207, 14/02/2022). Iron deficient subjects were selected to increase the probability of enrolling individuals that hyperabsorb iron^17^. Subjects (*n* = 8 per cohort in three ascending-dose cohorts) were entered into the study based on eligibility criteria and then randomized to either Arm 1 or Arm 2 in a 1:1 ratio. Subjects in Arm 1 received single doses of BBI-001 in Period 1 and placebo in Period 2; Subjects in Arm 2 received placebo in Period 1 and BBI-001 in Period 2. In Period 1 only, dosing in each cohort started with two sentinel subjects, with one subject each randomised to receive either BBI-001 or placebo. Sentinels were dosed 24 h prior to dosing the remaining subjects in each cohort (Fig. 2). Safety and tolerability were assessed according to the 2021 Medical Dictionary for Regulatory Activities (MedDRA). One subject was withdrawn from the study (investigator decision) after returning a positive test for cotinine after receiving one dose of BBI-001 in Period 1 and one subject was lost to follow-up after one dose of placebo in Period 1. The trial ended after 8 patients completed the each of the 3 dosing cohorts.

### Measurement of Iron absorption

Blood was collected immediately before dosing, as well as at 30 min, 1 h, 2 h, 4 h, 8 h, and 24 h post-dose after administration of each study drug (BBI-001 and placebo) and iron uptake was calculated. Iron absorption was quantified using an isotope ratio method for measuring iron uptake developed by Speich and colleagues^18,19^. The method was validated and analysis conducted by ALS Global.

## Results

### Study design and demographics

Demographics of the subject population are summarized in Table 1. Demographic factors were generally well-balanced between Arm 1 and Arm 2 and between all three doses. The starting BBI-001 dose level was 500 mg administered to Cohort 1 subjects. Subsequently, the BBI-001 dose levels increased to 1000 mg and 2000 mg in Cohort 2 and Cohort 3, respectively, based on a review of the prior cohort safety data by the Safety Review Committee. BBI-001 was given as a dry powder suspended with 60 mL of deionized water to form a suspension for ingestion. Approximately 150 mg of commercially available Original Fibre Metamucil^®^ (powdered psyllium, unflavored) served as the placebo in each dose cohort, which was chosen due to the similar color (tan to orange) of the suspension when compared to BBI-001 suspension. Immediately after BBI-001 or placebo was ingested, subjects were fed a standard low-iron meal supplemented with 10 mg of either ^57^Fe or ^58^Fe, respectively. Further details of the preparation of test articles and the isotope-containing meal are provided (Sup. File – Pharmacy Manual). After a washout period of at least one week, the subjects were given the crossover treatment and corresponding iron isotope in a blinded manner.

**Table 1:** Subject demographic parameters. sd = standard deviation; bmi = body mass index; a: BBI-001 administered in period 1, placebo administered in period 2; B: placebo administered in period 1, BBI-001 administered in period 2.

Demographic Parameter	Statistic	Sub-group	Cohort 1 Arm 1^a^ (*n* = 4)	Cohort 1 Arm 2^b^ (*n* = 4)	Cohort 2 Arm 1^a^ (*n* = 5)	Cohort 2 Arm 2^b^ (*n* = 4)	Cohort 3 Arm 1^a^ (*n* = 5)	Cohort 3 Arm 2^b^ (*n* = 5)	Total (*n* = 27)
Age (years)	Mean (SD)		32.0 (12.36)	33.3 (13.62)	30.4 (6.69)	27.5 (9.00)	22.2 (3.56)	25.4 (2.07)	28.2 (8.57)
Sex	n (%)	Female	4 (100%)	4 (100%)	5 (100%)	4 (100%)	5 (100%)	5 (100%)	27 (100%)
		Male	–	–	–	–	–	–	–
Ethnicity	n (%)	Hispanic or Latino	–	–	1 (20.0%)	1 (25.0%)	1 (20.0%)	–	3 (11.1%)
		Not Hispanic or Latino	4 (100%)	4 (100%)	4 (80.0%)	3 (75.0%)	4 (80.0%)	5 (100%)	24 (88.9%)
Race	n (%)	White	2 (50.0%)	2 (50.0%)	3 (60.0%)	4 (100%)	5 (100%)	5 (100%)	21 (77.8%)
		American Indian or Alaska Native	–	–	1 (20.0%)	–	–	–	1 (3.7%)
		Asian	1 (25.0%)	2 (50.0%)	1 (20.0%)	–	–	–	4 (14.8%)
		Native Hawaiian or Other Pacific Islander	1 (25.0%)	–	–	–	–	–	1 (3.7%)
Height (cm)	Mean (SD)		164.3 (8.66)	160.8 (6.24)	164.6 (2.61)	168.0 (9.20)	168.6 (5.46)	166.4 (6.73)	165.6 (6.51)
Weight (kg)	Mean (SD)		63.95 (7.980)	66.45 (8.972)	71.58 (12.049)	67.90 (11.502)	70.70 (8.356)	64.36 (13.167)	67.64 (10.041)
BMI (kg/m^2^)	Mean (SD)		23.873 (4.0093)	25.620 (1.6327)	26.350 (3.8547)	24.040 (3.2266)	25.054 (4.3572)	23.060 (2.8359)	24.683 (3.3429)

### Safety

BBI-001 was apparently safe and well tolerated in this study. There was no significant difference in safety between BBI-001 and placebo as assessed by clinical laboratory tests, vital signs, ECG assessments and physical examination findings. All reported treatment-emergent adverse events (TEAEs) reported were of mild severity and transient except for one possibly related TEAE classified as moderate severity somnolence in a subject receiving placebo (Table 2). No severe adverse event (SAE) was reported. None of these TEAEs were determined to be related to BBI-001. A summary of TEAEs is presented in Table 2. The most common TEAEs, reported by ≥ 5% subjects who received BBI-001 (at any dose) were upper respiratory tract infection, fatigue, and dysmenorrhea. A complete listing of TEAEs and AEs is available as Sup. Table 1.

One subject withdrew from the study after receiving one dose of BBI-001 (1000 mg) due to a cardiac-related treatment-emergent adverse event (TEAE) of “ECG QT prolonged”. The event was moderate in severity and considered unlikely related to study treatment since the QT prolongation also occurred on an ECG before the drug was taken. One subject was reported to have a TEAE of anemia when the subject received BBI-001 (500 mg). Considering all subjects were required to have iron-deficiency as an entry criteria, the attribution of this TEAE to BBI-001 was interpreted to be not related. No treatment-related TEAEs required medical intervention. There was one clinically significant physical examination finding related to a TEAE of viral URTI (moderate in severity and unlikely related to study treatment) when the subject was administered placebo.

**Table 2:** Treatment-related adverse events (TEAE). TEAEs deemed related to treatment were mild with the exception of one moderate TEAE (somnolence) in the placebo group. The TEAEs were consistent with this type of phase 1 study, similar between BBI-001 and placebo subjects, and with no predominant preferred term or dose correlation. TEAE = treatment-emergent adverse event; *TEAE classified as moderate in severity; a: BBI-001 administered in period 1, placebo administered in period 2; B: placebo administered in period 1, BBI-001 administered in period 2; C: probably related (patients were anemic as part of the enrollment criteria); D: possibly related.

Preferred term	Number (%) of subjects with related TEAEs [Number of related TEAEs reported]							
	Cohort 1 (500 mg BBI-001)		Cohort 2 (1000 mg BBI-001)		Cohort 3 (2000 mg BBI-001)		Placebo (*N* = 25)	Overall active BBI-001 (*N* = 26)
	Arm 1^a^ (*N* = 4)	Arm 2^b^ (*N* = 4)	Arm 1^a^ (*N* = 5)	Arm 2^b^ (*N* = 4)	Arm 1^a^ (*N* = 5)	Arm 2^b^ (*N* = 4)		
Anemia	1 (25.0%) [1]^c^	–	–	–	–	–	–	1 (3.8%) [1]^c^
Neutropenia	1 (25.0%) [1]^d^	–	–	–	–	–	–	1 (3.8%) [1]^d^
Abdominal discomfort	–	–	1 (20.0%) [1]^d^	–	–	–	–	1 (3.8%) [1]^d^
Fatigue		–	–	1 (25.0%) [1]^d^	–	–	1 (4.0%) [1]^d^	1 (3.8%) [1]^d^
Decreased appetite	–	–	–	–	–	–	1 (4.0%) [1]^d^	–
Headache	–	–	–	–		–	1 (4.0%) [1]^d^	–
Somnolence	–	–	–	–	–	–	1 (4.0%)* [1]^d^	–

### Efficacy

**Fig. 3:**
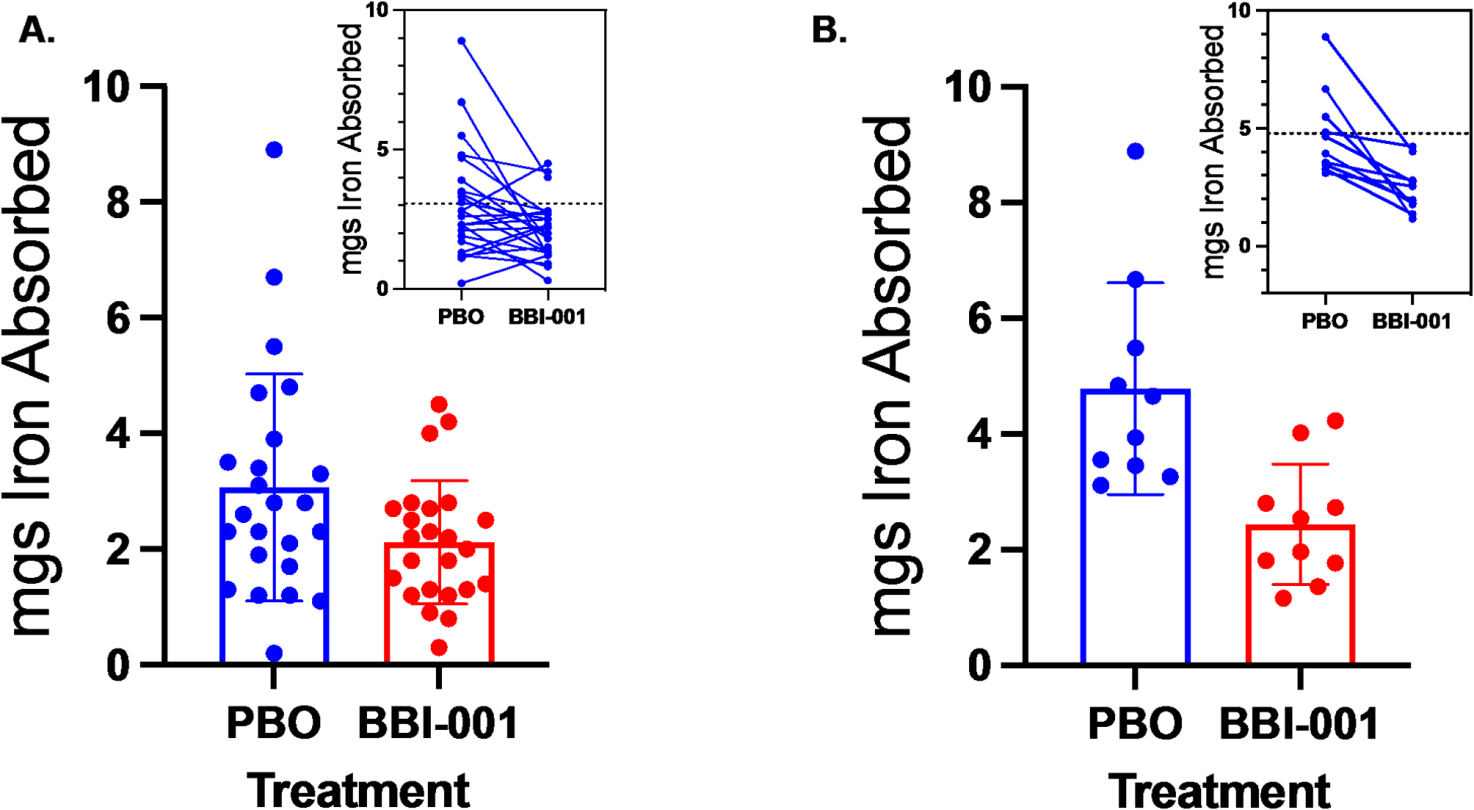
BBI-001 reduces iron isotope absorption. (**A**) BBI-001 significantly reduced iron absorption for all subjects compared to placebo (PBO), *p* < 0.05 (*p* = 0.018). (**B**) For individuals hyperabsorbing iron (> 3 mg iron absorbed on PBO), BBI-001 reduction of iron absorption was significant, *p* < 0.01 (*p* = 0.0023). Statistical analysis used a paired, two-tailed, t-test. Insets: Iron absorption per subject, placebo versus BBI-001 and mean change in iron absorbed.

**Fig. 4:**
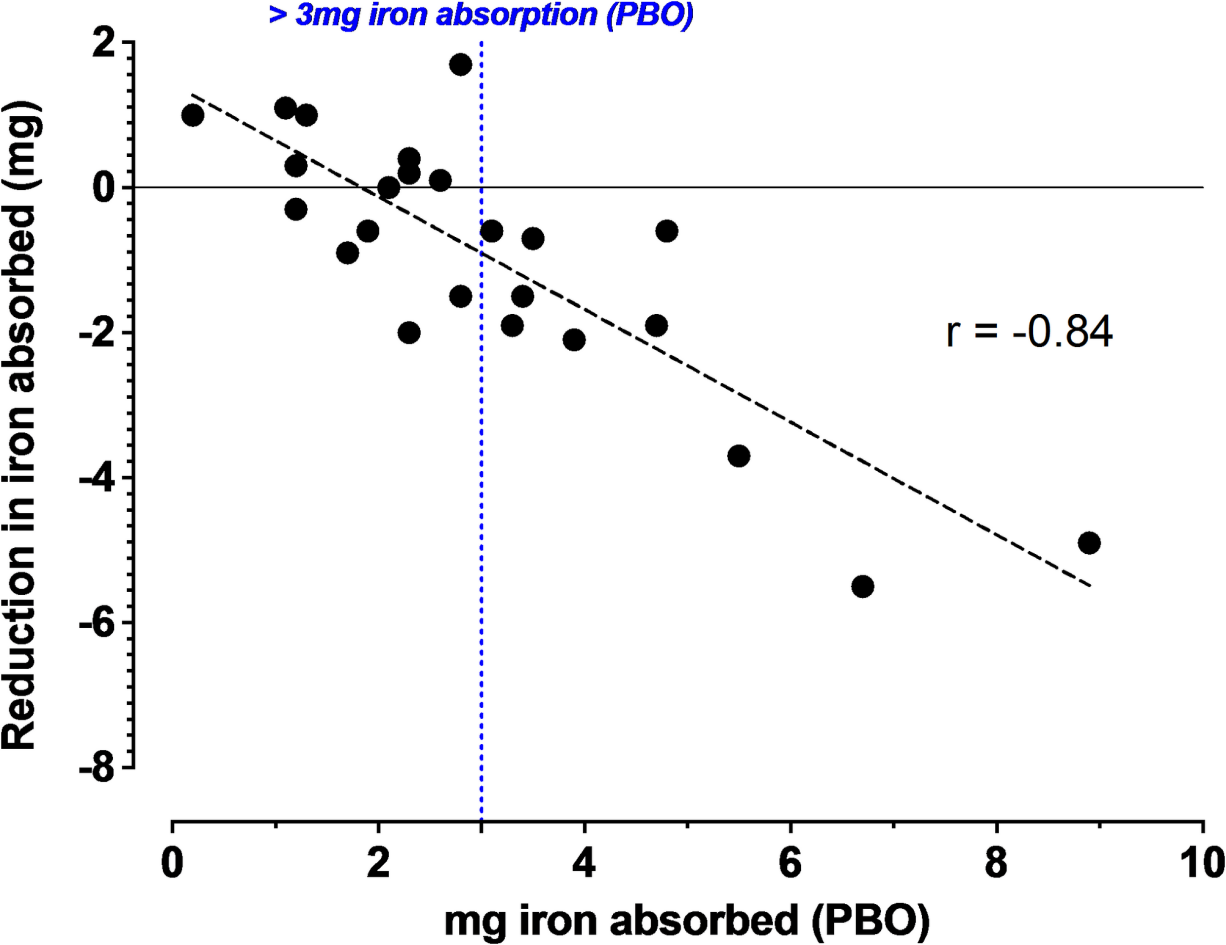
BBI0-001 is highly effective in subjects who hyperabsorbed iron on placebo. Correlation plot demonstrating a strong relationship between the mass of iron absorbed by patients taking placebo (PBO) versus the reduction in iron absorbed by the same patent taking BBI-001. Statistical analysis used a Pearson correlation, large strength of association defined as -0.5 to -1.0.

Anemic subjects were chosen for this study because about 30–40% of these subjects would be expected to hyperabsorb dietary iron at levels similar to HH patients^17^. BBI-001 significantly reduced the absorption of iron isotope compared to placebo across all subjects (*p* < 0.05, Fig. 3A). The total amount of iron absorbed were calculated from full pharmacokinetic curves (Sup. Figure 1), assuming the absorbed iron isotopes have the same elimination rate as iron sucrose^20^. Across all subjects, BBI-001 reduced iron absorption by 1 mg when compared to placebo. Subjects absorbing > 3 mg iron when taking placebo emulate the phenotype of ‘hyperabsorption’ observed in HH patients. Individuals absorbing > 3 mg of iron when taking placebo exhibited a 2.3 mg decrease in iron absorption when taking BBI-001 (*p* < 0.01, Fig. 3B) and these subjects consistently improved when crossing over to BBI-001 (Fig. 3B inset and Sup. Figure 2). In addition, the amount of iron that BBI-001 prevented from being absorbed was roughly proportional to the iron-avid state of the study subject with the greatest effect being seen in those with high iron absorption when given placebo (Fig. 4). Should this correlation persist in future studies, BBI-001 would be highly effective for patents most in need of iron absorption control.

## Discussion

Iron overload occurs in two ways: blood transfusion or iron accumulation from diet. Transfusion-dependent iron overload is associated with diseases such as beta thalassemia, where excess iron is present because of transfusions. These patients are not eligible for therapeutic phlebotomy because of underlying anemia and must have iron removed by systemic iron chelation. Systemic iron chelators, such as deferasirox, are prescribed when iron overload occurs in transfusion-dependent and certain non-transfusion-dependent thalassemias. Side effects, however, limit the use of deferasirox to these indications and it is rarely prescribed to treat iron overload in HH due to safety concerns^21^. In addition, long-term compliance with therapeutic phlebotomy to treat iron overload in HH can be less than 25% (https://pubmed.ncbi.nlm.nih.gov/14499790/).

BBI-001 was designed to provide a safe treatment for non-transfusion-dependent iron overload resulting from disorders such as HH. BBI-001 is an insoluble, crosslinked polymer particle large enough to remain in the gastrointestinal tract. Restricting a drug to the gut mitigates the risk of systemic toxicity. In fact, BBI-001 was well tolerated in this trial and prior animal studies^16^. Other similar non-absorbed therapeutics, such as sevelamer or sodium zirconium cyclosilicate, can exhibit gastrointestinal side effects, such as bowel obstruction, constipation or diarrhea^22,23^. However, the insoluble BBI-001 particles have negligible osmotic effects, which may explain why local gastrointestinal side effects were not observed in this trial or in two-week, multiple ascending dose toxicity studies in rats and dogs. An upcoming clinical trial will determine if multiple ascending doses in patients show a similar low frequency of side effects.

BBI-001 was designed to rapidly and effectively bind iron in the gut prior to absorption. Mimicking the structure of the natural siderophore enterobactin endows BBI-001 with high avidity and selectivity for iron^16^ The compound exhibits the highest reported binding constant for iron of any synthetic material^13,14^. To establish proof-of-mechanism, we probed the ability of BBI-001 to prevent iron absorption by employing traceable, non-radioactive iron isotopes supplemented in a standardized breakfast. After administration, BBI-001 particles mix with ingested food and capture the digested iron before it can be absorbed by the dimetallic transporter 1 at the intestinal wall. Here, only a single dose was studied, therefore, future studies will explore multiple doses of BBI-001 with meals.

BBI-001 effectively reduced the absorption of iron isotopes supplemented in breakfast meals when comparing all dose levels of drug to powdered psyllium placebo. Ten (10) out of the 24 anemic subjects absorbed ~ 3–9 mg of the 10 mg of iron isotope administered and were defined as iron hyperabsorbers. These individuals emulated the excessive iron absorption of patients with HH who would require maintenance phlebotomy. BBI-001 was highly effective in hyperabsorbers, reducing average the iron absorption by ~ 6 mg in the most iron-avid participant. A strong correlation was identified demonstrating BBI-001 capacity to bind the most iron in subjects who absorbed the most iron when taking placebo (*r* = -0.84).

## Conclusions

BBI-001 effectively and safely blocked the absorption of dietary iron in a double-blind, placebo-controlled Phase 1b crossover clinical trial in patients with iron deficiency. Single dose administrations of BBI-001 were safe and well tolerated up to the maximum dose of 2000 mg. BBI-001 significantly reduced the absorption of iron isotopes compared to a placebo control in the same subject. High potency was demonstrated in subjects that absorbed the most iron isotope on placebo. BBI-001 may offer a safe and effective pharmaceutical strategy for reducing dietary iron absorption as an alternative to life-long therapeutic phlebotomy.

## Electronic supplementary material

Below is the link to the electronic supplementary material.

Supplementary Material 1

Supplementary Material 2

Supplementary Material 3

## Data Availability

Relevant data generated or analyzed during this study are included in this published article and its supplementary information.
