# Supplementary material for: A phase 1b randomised clinical trial evaluating BBI-001, a non-absorbed oral therapeutic for the treatment of iron overload
Source: Sci Rep. 2025 May 17;15:17210. doi: 10.1038/s41598-025-01421-4 (PMC12085664; doi:10.1038/s41598-025-01421-4)
Supplement: Supplementary file 1 — Supplementary Material 1 [file 41598_2025_1421_MOESM1_ESM.docx]

**Supplementary Tables and Figures**

**Supplementary Table 1.** A full listing of related and unrelated TEAEs and AEs is shown. A dose related response was not noted. The one TEAE leading to study discontinuation was due to QTc prolongation on the ECG prior to treatment. Abbreviations: TEAE = treatment-emergent adverse event; SAE = serious adverse event; a: BBI-001 administered in Period 1, placebo administered in Period 2; b: Placebo administered in Period 1, BBI-001 administered in Period 2; *If a subject had multiple occurrences of a TEAE, the subject is presented once in the subject count of maximum relationship to study drug for a System Organ Class and Preferred Term.

|  | Number (%) of Subjects with TEAEs  [Number of TEAEs Reported] | | | | | | | |
| --- | --- | --- | --- | --- | --- | --- | --- | --- |
|  | Cohort 1 (500 mg BBI-001) | | Cohort 2 (1000 mg BBI-001) | | Cohort 3 (2000 mg BBI-001) | | Placebo (N=25) | Overall  Active  BBI-001 (N=26) |
|  | Arm 1^a^ (N=4) | Arm 2^b^ (N=4) | Arm 1^a^ (N=5) | Arm 2^b^ (N=4) | Arm 1^a^ (N=5) | Arm 2^b^ (N=4) |  |  |
| TEAEs | 4 (100%) [7] | 1 (25.0%) [2] | 2 (40.0%) [3] | 2 (50.0%) [2] | 3 (60.0%) [6] | 2 (50.0%) [2] | 9 (36.0%) [12] | 14 (53.8%) [22] |
| Mild TEAEs | 4 (100%) [7] | 1 (25.0%) [2] | 1 (20.0%) [2] | 2 (50.0%) [2] | 1 (20.0%) [4] | 2 (50.0%) [2] | 8 (32.0%) [11] | 11 (42.3%) [19] |
| Moderate TEAEs | - | - | 1 (20.0%) [1] | - | 1 (20.0%) [1] | - | 1 (4.0%) [1] | 2 (7.7%) [2] |
| Severe TEAEs | - | - | - | - | 1 (20.0%) [1] | - | - | 1 (3.8%) [1] |
| SAEs | - | - | - | - | - | - | - | - |
| Unrelated TEAEs | 1 (25.0%) [1] | 1 (25.0%) [2] | 1 (20.0%) [1] | 1 (25.0%) [1] | -*  [2] | - | 5 (20.0%) [7] | 4 (15.4%) [7] |
| Unlikely Related TEAEs | 1 (25.0%) [4] | - | -*  [1] | - | 3 (60.0%) [4] | 2 (50.0%) [2] | 1 (4.0%) [1] | 6 (23.1%) [11] |
| Possibly Related TEAEs | 1 (25.0%) [1] | - | 1 (20.0%) [1] | 1 (25.0%) [1] | - | - | 3 (12.0%) [4] | 3 (11.5%) [3] |
| Probably Related TEAEs | 1 (25.0%) [1] | - | - | - | - | - | - | 1 (3.8%) [1] |
| Definitely Related TEAEs | - | - | - | - | - | - | - | - |
| TEAEs leading to study discontinuation | - | - | 1 (20.0%) [1] | - | - | - | - | 1 (3.8%) [1] |
| TEAEs not recovered/not resolved | 1 (25.0%) [1] | - | - | - | - | - | 1 (4.0%) [1] | 1 (3.8%) [1] |

**Supplementary Figure 1.** Sample iron isotope absorption pharmacokinetics between Placebo and BBI-001 treatments for ‘high absorber’ (A) exhibiting iron hyperabsorption and ‘low absorber’ (B) individuals with normal iron absorption.

**Supplementary Figure 2.** Iron isotope absorption comparison between placebo and BBI-001 across dose cohorts 500 mg (A), 1000 mg (B), and 2000 mg (C).
