# Supplementary material for: A phase 1b randomised clinical trial evaluating BBI-001, a non-absorbed oral therapeutic for the treatment of iron overload
Source: Sci Rep. 2025 May 17;15:17210. doi: 10.1038/s41598-025-01421-4 (PMC12085664; doi:10.1038/s41598-025-01421-4)
Supplement: Supplementary file 2 — Supplementary Material 2 [file 41598_2025_1421_MOESM2_ESM.docx]

# Pharmacy and Meal Preparation

## IP Dispensing

BBI-001 and (Placebo) Metamucil^®^ doses were prepared as above by unblinded pharmacy staff to preserve study blinding. Fe isotopes were supplied in individual 2 ml aliquots of 10 mg ready for use and were prepared with a standardized meal for administration.

## BBI-001

Pharmacy preparation protocol (completed by unblinded Pharmacy Team)

1. Open the bulk container of BBI-001.
2. Weigh the required quantity of BBI-001 (+/- 5% weight of the required dose) for each subject into a suitable airtight container. Minimize exposure to open air as much as possible.

Clinic

1. Add 60 ml of deionized bottled water to the IP containing container and swirl with a spoon to disperse.
2. Administer within 5 minutes after addition of water.
3. Add an additional 60 ml of deionized bottled water to the drinking glass for rinsing to ensure the complete dose is taken.

Metamucil® - Natural granular smooth powder

Pharmacy preparation protocol

1. Weigh 150 mg of Metamucil^®^ powder (purchased in Australia) into a suitable container
2. Add 60 mL of deionized bottled water to the Metamucil containing container and swirl with a spoon to disperse.
3. Administer within 5 minutes after addition of water.
4. Add an additional 60 ml of deionized bottled water to the drinking glass for rinsing to ensure the complete dose is taken.

### Fe Isotopes and Meal

The product was supplied as individual aliquots containing 10 mg of Fe57 or Fe58 isotope. The Fe Isotope expiry was 24 hours post removal from the fridge, provided it was kept below 25°C. The isotope solution containers were opened with pliers which were provided with the containers.

The isotopic solution was given along with standardized meals as follows:

The standardized breakfast meal consisted of two slices of non-iron fortified white toast bread. The toast bread was served on a plate or bowl, and on one slice of toast, 10 g of pre-packaged honey was added, while on the other slice of toast the isotopic solution was sprinkled quantitatively using a disposable Pasteur pipette. The container holding the isotopic solution was rinsed with 3 x 1 ml of de-ionised bottled water, and the subsequent rinsing solution was added to the toast bread. The toast bread was then folded as a sandwich and served to the subjects, who consumed it using a knife and a fork (to avoid spilling isotopic solution onto their hands). The plate or bowl was rinsed with 2x10 ml of de-ionized bottled water and the water was consumed by the participant. Participants were informed prior to the meal of the need to consume the meal in full (in its entirety) and of the need to rinse the plate or bowl with bottled water to ensure quantitative administration. Participants received 10 mg of isotopic solution on the standard breakfast.

Food and Water Restrictions:

- Fasted from food from 9 pm until 4 hours post dose. (Except Fe Isotope meal)
- Water restricted from 12 am. No water restriction post dose

## IP Compliance

During this clinical trial, compliance was monitored by site staff witnessing of dosing and documentation in participant study file. Staff ensured that the complete doses of the IP, Placebo and Fe isotopes were taken by following the specific administration procedures (rinsing with water) as above.
